# Association between mucosectomy and endoscopic outcomes in patients with ileal pouch–anal anastomosis

**DOI:** 10.1093/gastro/goad078

**Published:** 2024-07-04

**Authors:** Amy Hembree, Bo Shen, Daniel Freedberg

**Affiliations:** Division of Digestive and Liver Diseases, Department of Medicine, Columbia University Irving Medical Center, New York-Presbyterian Hospital, New York, NY, USA; Division of Digestive and Liver Diseases, Department of Medicine, Columbia University Irving Medical Center, New York-Presbyterian Hospital, New York, NY, USA; Division of Digestive and Liver Diseases, Department of Medicine, Columbia University Irving Medical Center, New York-Presbyterian Hospital, New York, NY, USA

**Keywords:** ileal pouch–anal anastomosis, IPAA, pouchitis, cuffitis, inflammatory bowel disease, IBD

## Abstract

**Background:**

In patients with inflammatory bowel disease (IBD) for whom medical therapy is unsuccessful or who develop colitis-associated neoplasia, restorative proctocolectomy with ileal pouch–anal anastomosis (IPAA) is often indicated. One consideration for surgeons performing this procedure is whether to create this anastomosis using a stapled technique without mucosectomy or using a hand-sewn technique with mucosectomy. This study tested the association between IPAA anastomosis technique and cuffitis and/or pouchitis, assessed endoscopically.

**Methods:**

This was a retrospective cohort study. We included consecutive adult patients with IBD who had undergone IPAA and had received index pouchoscopies at Columbia University Irving Medical Center between 2020 and 2022. Patients were then followed up from this index pouchoscopy for ≤12 months to a subsequent pouchoscopy. The primary exposure was mucosectomy vs non-mucosectomy and the primary outcome was cuffitis and/or pouchitis, defined as a Pouch Disease Activity Index endoscopy subscore of ≥1.

**Results:**

There were 76 patients who met study criteria including 49 (64%) who had undergone mucosectomy and 27 (36%) who had not. Rates of cuffitis and/or pouchitis were 49% among those with mucosectomy vs 41% among those without mucosectomy (*P *=* *0.49). Time-to-event analysis affirmed these findings (log-rank *P *=* *0.77). Stricture formation was more likely among patients with mucosectomy compared with those without mucosectomy (45% vs 19%, *P *=* *0.02).

**Conclusions:**

There was no association between anastomosis technique and cuffitis and/or pouchitis among patients with IBD. These results may support the selection of stapled anastomosis over hand-sewn anastomosis with mucosectomy.

## Introduction

In patients with inflammatory bowel disease (IBD) for whom medical therapy is unsuccessful or who develop neoplasia, restorative proctocolectomy with ileal pouch–anal anastomosis (IPAA) is often indicated, as opposed to standard colectomy with permanent end ileostomy, as it preserves the natural defecation route and circumvents the need for a permanent abdominal stoma [1, 2]. Although this procedure often leads to symptoms and quality-of-life improvement, it may be associated with complications, including inflammation of the pouch or rectal cuff, known as “pouchitis” and “cuffitis,” respectively [1, 3].

When performing a restorative proctocolectomy using IPAA, surgeons use distal rectal tissue to join the pouch to the anus, thus forming a “rectal cuff” above the anal transition zone. One consideration for surgeons performing this procedure is determining whether to create this anastomosis using a stapled technique without mucosectomy or a hand-sewn technique with mucosectomy.

Although stapled anastomosis is often the preferred method due to improved nocturnal continence compared with hand-sewn anastomosis [4] as well as the convenience of a faster procedure, this technique requires a minimum of 1.5–2 cm of the rectal cuff, increasing the quantity of remnant rectal tissue susceptible to residual ulcerative colitis (UC) [2]. Hand-sewn anastomosis with mucosectomy, on the other hand, does not have such stringent minimal length requirements and more anorectal epithelia is able to be resected [5, 6]. But hand-sewn anastomosis is a more technically challenging and time-consuming procedure, and leads to small islands of rectal mucosa remaining that may later become susceptible to inflammation in the form of cuffitis, despite all attempts to completely remove rectal tissue [7, 8]. As with the stapled anastomosis, the remnant rectal tissue is susceptible to residual UC, leading to cuffitis [2].

In theory, performing a mucosectomy would leave less rectal tissue susceptible to dysplasia or inflammation, thereby reducing the risk of cancer or cuffitis. However, recent evidence has shown that stapled anastomosis has similar risk of dysplasia compared to hand-sewn anastomosis [9]. There is less available literature on the association of mucosectomy with cuffitis. One meta-analysis commented on the difficulty of determining whether or not mucosectomy would reduce the occurrence of cuffitis given that it so often co-occurs with pouchitis, though they note that intuition would suggest that this is the case [10].

In this study, we examine the association of mucosectomy with cuffitis and pouchitis. While other studies have focused on several outcomes associated with mucosectomy, to our knowledge, ours is the first retrospective cohort study to characterize pouch and cuff endoscopic and histologic outcomes associated with mucosectomy.

## Methods

### Patient population

Manual chart review was conducted for all adult patients with IBD who had undergone IPAA and who had received pouchoscopies at Columbia University Irving Medical Center between 2020 and 2022. Institutional review board approval for this study was granted (approval number AAAU2498) and informed consent was waived.

### Inclusion and exclusion criteria

**Figure 1.**
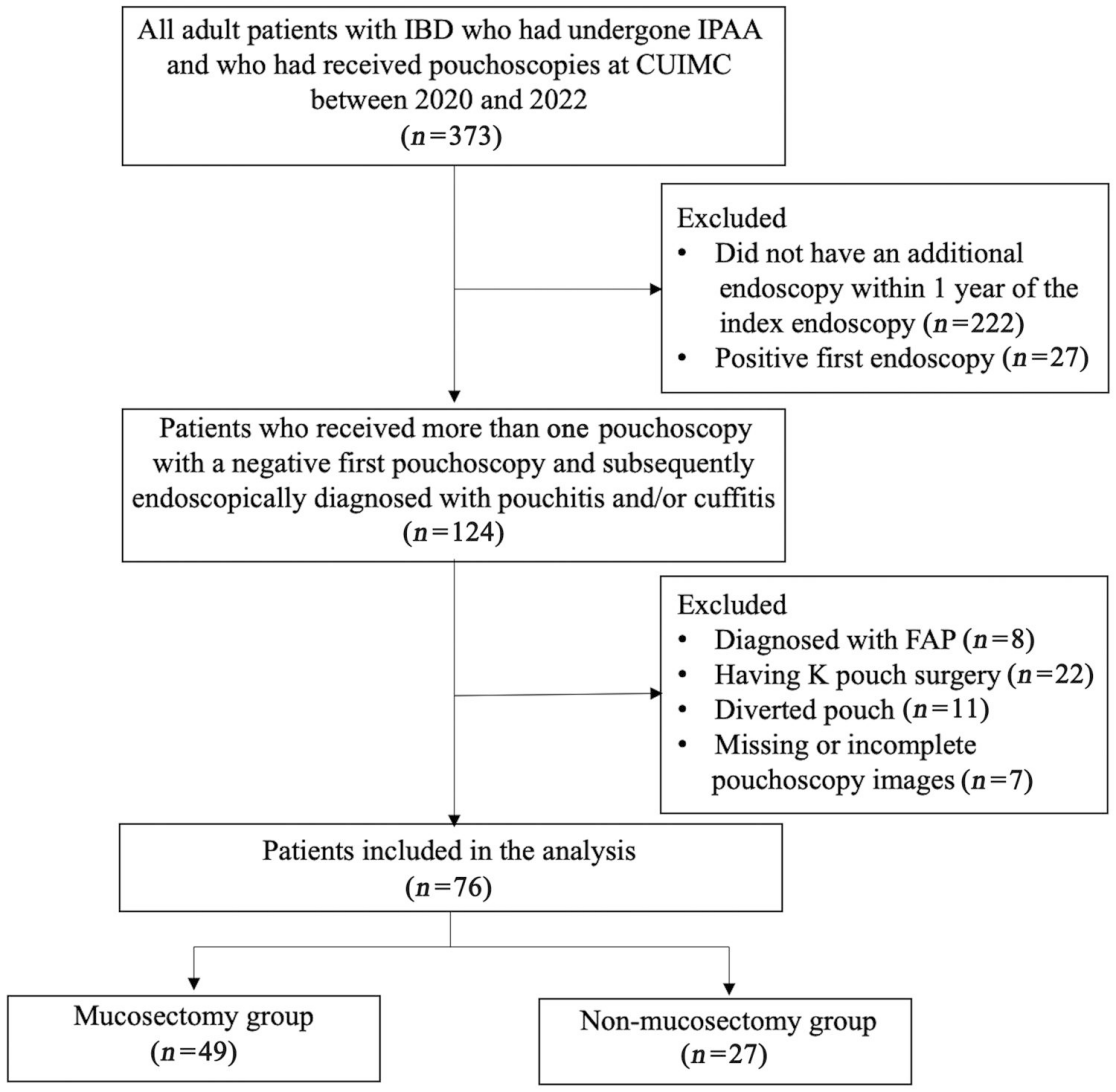
Flow chart of patient assignment in this study. CUIMC = Columbia University Irving Medical Center, IBD = inflammatory bowel disease, FAP = familial adenomatous polyposis.

For each patient, we reviewed the first endoscopy available during the study period for the presence of cuff or pouch inflammation. This was defined as use of the words “inflammation,” “pouchitis,” “cuffitis,” or a listed Pouch Disease Activity Index (PDAI) score. Patients without cuffitis or pouchitis were considered to have a negative index endoscopy and were included in the study, as were patients with an additional endoscopy within 1 year of the index endoscopy. Additionally, the exclusion criteria were as follows: (i) patients with familial adenomatous polyposis, (ii) those who had K pouch surgery, (iii) those with diverted pouch, and (iv) those whose pouchoscopy images were missing or incomplete (Figure 1).

### Study design

This was a retrospective cohort study. Patients were followed up from the index pouchoscopy, when cuffitis or pouchitis was absent, to the next pouchoscopy. Based on the findings at the time of the second pouchoscopy, the presence or absence of cuffitis and/or pouchitis, the primary outcome of this study, was noted.

### Primary exposure

The primary exposure was mucosectomy, defined based on the follow-up pouchoscopy report, which was performed within 1 year of the index pouchoscopy based on the study design (Supplementary Figure 1). For patients in which the presence or absence of mucosectomy was not clearly delineated in the written report, an expert clinician (B.S.) manually re-reviewed the endoscopy imaging to make this determination.

### Primary outcome

The primary outcome was cuffitis and/or pouchitis, classified as present vs absent at the time of the second pouchoscopy, which was performed within 1 year of the index endoscopy. Pouchoscopies were manually scored by the expert clinician (B.S.) using the PDAI scoring system. This scoring system assesses for six components: edema, granularity, friability, loss of vascular pattern, mucous exudates, and ulcerations, with 1 point given for the presence of each of these features. Pouchitis and cuffitis were defined as PDAI scores of ≥1 in the pouch or cuff, respectively.

### Secondary outcomes

In addition to the primary outcome of cuffitis and/or pouchitis, other endoscopic outcomes were assessed through expert review of endoscopy reports. These outcomes included stricture, pouch prolapse, afferent limb syndrome, pouch leak, pouch polyp, pouch dysplasia/cancer, and perianal dermatitis. Concurrent pouch therapy included stricture dilation, stricturotomy, sinusotomy, banding of prolapse, polypectomy, and fistulotomy. Any evidence of inflammation on histology, including polymorphic nuclear leukocyte infiltration or ulceration, was considered at least mild inflammation.

### Co-variables

We gathered patients' baseline characteristics, surgical variables, comorbidities, and medication use at the time of index endoscopy. Age at the time of index endoscopy was divided into three categories: 18–50, 51–70, and >70 years old. Smoking status was classified as never smoker, former smoker, or current smoker at the time of index endoscopy. Body mass index was recorded and divided into four categories: <18.5, 18.5–24.9, 25.0–29.9, and ≥30 kg/m^2^. Family history of IBD or colon cancer included parents, grandparents, siblings, or children. IBD subtype classified patients as having UC, Crohn’s disease, or indeterminate based on clinical notes. Years since IBD diagnosis, defined as the time between initial diagnosis and index endoscopy, was classified into four categories: 0–9.9, 10.0–19.9, 20.0–29.9, and ≥30 years. Surgical stage refers to whether the IPAA procedure was performed in one, two, or three operations or “stages.” Indication for colectomy was classified as either medically refractory disease or neoplasia. Comorbidities listed in the medical record were assessed at the time of index endoscopy and were classified as present or absent, including coronary artery disease, previous coronary artery bypass graft, chronic obstructive pulmonary disease, pulmonary embolism, deep vein thrombosis, chronic kidney disease, and end-stage renal disease. Medication use at time of index pouchoscopy included antibiotics, topical or oral mesalamine, topical or oral steroids, mercaptopurine/azathioprine/methotrexate, tumor necrosis factor blockers, vedolizumab, ustekinumab, and other disease-modifying agents. Patients were classified as taking these medications if documentation of medication use was present in the medical record at the time of index pouchoscopy.

### Statistical analysis

Data are expressed as frequencies and percentages for categorical variables, and were compared using the chi-squared test. Data are expressed as median (interquartile range) for continuous variables and were compared using the two-sample Wilcoxon rank-sum (Mann–Whitney) test. Univariable and multivariable logistic regression was performed for the primary outcome (presence of cuffitis and/or pouchitis). Variables were considered for inclusion in the final multivariable model if they had a univariable relationship (*P *<* *0.10) with either the primary exposure or the primary outcome. Time-to-event analysis was conducted for the primary outcome. Analysis was conducted using Stata version 14.2 (StataCorp LLC, Texas, USA).

## Results

### Patient population

A total of 373 adult patients with IBD who had undergone IPAA and who had received pouchoscopies during the study period were identified, of whom 76 patients met the study criteria and were divided into two groups, i.e. those who had undergone mucosectomy (*n *=* *49) and those who had not (*n *=* *27).

### Baseline characteristics

Clinical characteristics of patients are shown in Table 1. In this study, 52.6% of the patients were male and most were diagnosed with IBD 10–30 years ago (55.7%). The majority of patients were 18–50 years old (52.6%), non-smokers (82.7%), and had a body mass index of 18.5–24.9 kg/m^2^ (60.5%); 34.2% of patients had a family history of IBD or colon cancer and 75.0% of patients had an IBD subtype of UC. There was no difference in these characteristics between the mucosectomy and non-mucosectomy groups. Mucosectomy was associated with increased time since IBD diagnosis (*P *=* *0.04), being male (*P *=* *0.04), and antibiotic use (*P *=* *0.02). There was no difference in medication regimen at the time of index endoscopy nor the incidence of coronary artery disease, chronic obstructive pulmonary disease, liver cirrhosis, deep vein thrombosis, pulmonary embolism, or chronic kidney disease/end-stage renal disease between the mucosectomy and non-mucosectomy groups. Time between endoscopies was a median of 7 months with no difference found between the mucosectomy and non-mucosectomy groups.

**Table 1.** Comparison of clinical characteristics at the time of the index pouchoscopy between the mucosectomy group and the non-mucosectomy group

Variable	All	Mucosectomy	Non-mucosectomy	*P*-value
	(*n *=* *76)	(*n *=* *49)	(*n *=* *27)	
Demographic				
Age, years				0.82
18–50	40 (52.6)	26 (53.1)	14 (51.9)	
51–70	32 (42.1)	21 (42.8)	11 (40.7)	
>70	4 (5.3)	2 (4.1)	2 (7.4)	
Male	40 (52.6)	30 (61.2)	10 (37.0)	0.04
Smoking status^a^				0.75
Non-smoker	62 (82.7)	39 (81.3)	23 (85.2)	
Former smoker	12 (16.0)	8 (16.7)	4 (14.8)	
Current smoker	1 (1.3)	1 (2.1)	0 (0.0)	
Body mass index, kg/m^2^				0.92
<18.5	7 (9.2)	4 (8.2)	3 (11.1)	
18.5–24.9	46 (60.5)	31 (63.3)	15 (55.6)	
25.0–29.9	20 (26.3)	12 (24.5)	8 (29.6)	
≥30	3 (3.9)	2 (4.1)	1 (3.7)	
Family history of IBD or colon cancer^b^	25 (34.2)	16 (34.8)	9 (33.3)	0.42
IBD information				
IBD subtype				0.59
Crohn's disease	10 (13.2)	7 (14.3)	3 (11.1)	
Ulcerative colitis	57 (75.0)	35 (71.4)	22 (81.5)	
Indeterminate	9 (11.8)	7 (14.3)	2 (7.4)	
Time since IBD diagnosis^c^, years				0.04
0–9.9	10 (19.2)	3 (9.7)	7 (33.3)	
10.0–19.9	15 (28.8)	9 (29.0)	6 (28.6)	
20.0–29.9	14 (26.9)	12 (38.7)	2 (9.5)	
≥30	13 (25.0)	7 (22.6)	6 (28.6)	
Time between endoscopies, months, median [IQR]	7 [4, 9]	7 [2, 9]	7 [5.5, 8]	0.64
Surgical information				
J pouch^d^	71 (95.9)	45 (93.8)	26 (100.0)	0.39
Time since surgery^d^, years				0.01
0–10	25 (33.8)	9 (19.1)	16 (59.3)	
10–20	20 (27.0)	16 (34.0)	4 (14.8)	
20–30	18 (24.3)	15 (31.9)	3 (11.1)	
>30	11 (14.9)	7 (14.9)	4 (14.8)	
Surgical stage^e^				0.12
Stage 1	6 (17.6)	3 (15.0)	3 (21.4)	
Stage 2	12 (35.3)	10 (50.0)	2 (14.3)	
Stage 3	16 (47.1)	7 (35.0)	9 (64.3)	
Indication for surgery				0.32
Medically refractory disease	70 (92.1)	44 (89.8)	26 (96.3)	
Cancer	6 (7.9)	5 (10.2)	1 (3.7)	
Medical history				
Coronary artery disease	1 (1.3)	0 (0.0)	1 (3.7)	0.18
Chronic obstructive pulmonary disease	3 (3.9)	1 (2.0)	2 (7.4)	0.25
Coronary artery bypass graft	0 (0.0)	0 (0.0)	0 (0.0)	ns
Liver cirrhosis	1 (1.3)	0 (0.0)	1 (3.7)	0.18
History of clotting				0.12
Pulmonary embolism	2 (2.6)	2 (4.1)	0 (0.0)	
Deep vein thrombosis	5 (6.6)	5 (10.2)	0 (0.0)	
Chronic kidney disease/end-stage renal disease	1 (1.3)	1 (2.0)	0 (0.0)	0.46
Current medication use				
Antibiotic	19 (25.0)	8 (16.3)	11 (40.7)	0.02
Topical or oral mesalamine	14 (18.4)	9 (18.4)	5 (18.5)	0.99
Topical or oral steroids	9 (11.8)	4 (8.2)	5 (18.5)	0.18
6MP/AZA/MTX	1 (1.3)	1 (2.0)	0 (0.0)	1.00
Anti-TNFs	9 (11.8)	6 (12.2)	3 (11.1)	0.88
Vedolizumab	9 (11.8)	4 (8.2)	5 (18.5)	0.18
Ustekinumab	9 (11.8)	5 (10.2)	4 (14.8)	0.55
Other disease-modifying agents	1 (1.3)	1 (2.0)	0 (0.0)	1.00

IBD = inflammatory bowel disease; IQR = interquartile range; 6MP = 6-mercaptopurine; AZA = azathioprine; MTX = methotrexate; Anti-TNFs = antitumor necrosis factor; ns = not significant given small sample size.

a *n *=* *75.

b *n *=* *73.

c *n *=* *52.

d *n *=* *74.

e *n *=* *34.

### Surgical characteristics

The majority of patients had a J pouch (95.9%), as opposed to an S pouch; the most common indication was medically refractory disease (92.1%) and the most common surgical approach was three-stage (47.1%) (Table 1). There was no statistically significant difference between the mucosectomy and non-mucosectomy groups in pouch type, indication for surgery, or surgical stage (all *P *>* *0.05). Mucosectomy was associated with increased time since surgery (*P *=* *0.01).

### Clinical outcomes

The overall incidence of cuffitis or pouchitis was 46.0% among all patients. There was no association between mucosectomy and cuffitis and/or pouchitis (*P *=* *0.49) (Table 2). Analysis stratified by antibiotic use also did not show any association between mucosectomy and cuffitis and/or pouchitis (Supplementary Table 1). Stricture was observed in 44.9% of those who had mucosectomy vs 18.5% of those who did not (*P *=* *0.02). There was no association between mucosectomy status and other pouch complications including pouch prolapse, leak, polyps, dysplasia, afferent limb syndrome, or perianal dermatitis.

**Table 2.** Comparison of outcomes at the time of the second pouchoscopy between the mucosectomy group and the non-mucosectomy group

Variable	All	Mucosectomy	Non-mucosectomy	*P*-value
	(*n *=* *76)	(*n *=* *49)	(*n *=* *27)	
Cuffitis	26 (34.2)	16 (32.7)	10 (37.0)	0.70
Cuff PDAI score, median [IQR]	0 [0, 3]	0 [0, 2]	0 [0, 3]	0.91
Pouchitis	21 (27.6)	15 (30.6)	6 (22.2)	0.43
Pouch PDAI score, median [IQR]	0 [0, 1]	0 [0, 1]	0 [0, 1]	0.53
Cuffitis and/or pouchitis	35 (46.1)	24 (49.0)	11 (40.7)	0.49
Stricture	27 (35.5)	22 (44.9)	5 (18.5)	0.02
Stricture at anastomosis	17 (22.4)	15 (30.6)	2 (7.4)	0.02
Stricture at inlet	16 (21.1)	12 (24.5)	4 (14.8)	0.32
Stricture at stoma closure site	1 (1.3)	1 (2.0)	0 (0.0)	ns
Pouch prolapse	28 (36.8)	17 (34.7)	11 (40.7)	0.60
Afferent limb syndrome	0 (0.0)	0 (0.0)	0 (0.0)	ns
Pouch leak	0 (0.0)	0 (0.0)	0 (0.0)	ns
Pouch polyp	3 (3.9)	1 (2.0)	2 (7.4)	0.25
Pouch dysplasia/cancer	0 (0.0)	0 (0.0)	0 (0.0)	ns
Concurrent pouch therapy	49 (64.5)	34 (69.4)	15 (55.6)	0.23
Perianal dermatitis	2 (2.6)	2 (4.1)	0 (0.0)	0.29
Histologic outcomes^a^				ns
Cuffitis	9 (69.2)	5 (62.5)	4 (80.0)	
Pouchitis	9 (69.2)	5 (62.5)	4 (80.0)	
Afferent limb inflammation	4 (30.8)	3 (37.5)	1 (20.0)	

PDAI = Pouch Disease Activity Index; IQR = interquartile range; ns = not significant given small sample size.

a *n *=* *13.

**Figure 2.**
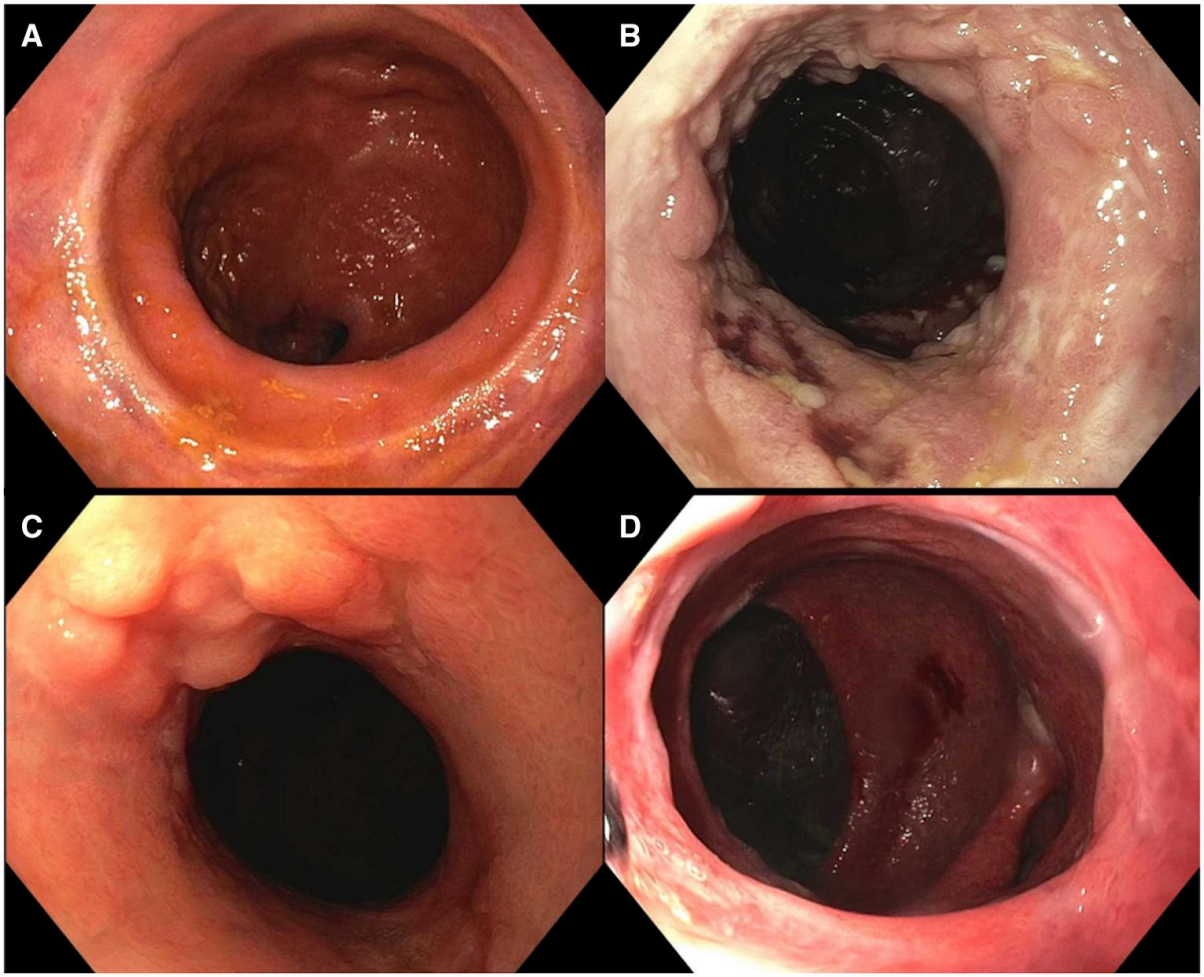
Endoscopic images of the rectal cuff in patients with IPAA. (A) Non-mucosectomy cuff without inflammation. (B) Non-mucosectomy cuff with cuffitis. (C) Mucosectomy cuff with evidence of cuffitis (anal transition zone nodularity). (D) Mucosectomy cuff without inflammation.

**Figure 3.**
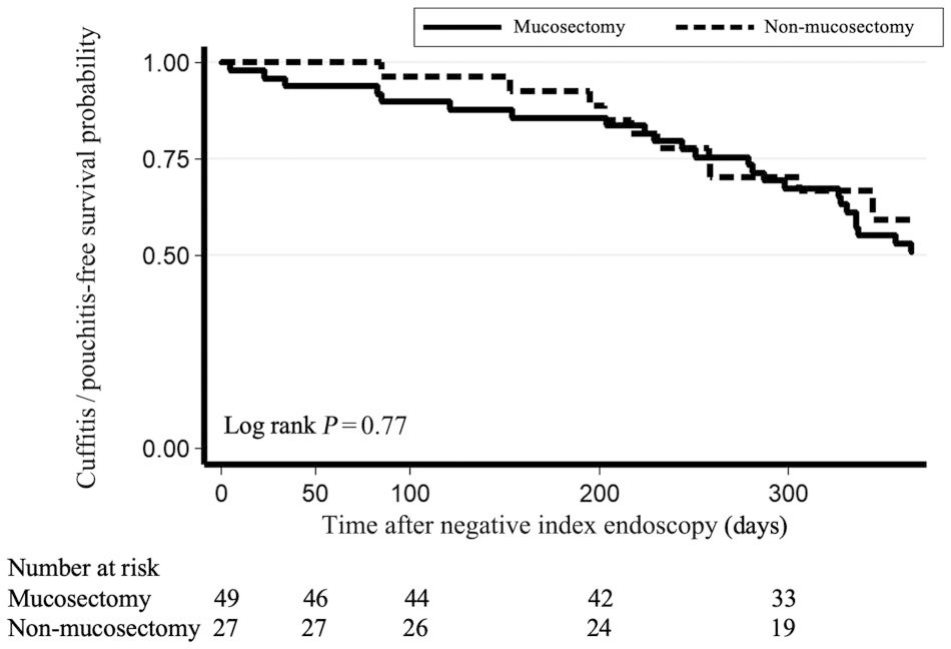
Time-to-event analysis for the outcome of no cuffitis or pouchitis. No significant difference was found between patients who had undergone mucosectomy and those who had undergone non-mucosectomy with respect to the primary outcome of cuffitis and/or pouchitis.

Histology was available for only a small subset of patients (*n *=* *13), as biopsy is often avoided out of concern for patient discomfort, and thus comparisons between two groups were not deemed informative (Table 2). Examples of endoscopic findings demonstrating non-mucosectomy cuff without inflammation (Figure 2A), non-mucosectomy cuff with cuffitis (Figure 2B), mucosectomy cuff with evidence of cuffitis (anal transition zone nodularity) (Figure 2C), and mucosectomy cuff without inflammation (Figure 2D) are shown. On time-to-event analysis for the outcome of cuffitis and/or pouchitis during the 1-year study period, there was no difference between groups (log-rank *P *=* *0.77) (Figure 3).

When stratified by the primary outcome of cuffitis and/or pouchitis, only smoking status was associated with cuffitis and/or pouchitis (*P *=* *0.02) (Table 3). There were no other differences between those who did vs those who did not have cuffitis or pouchitis at the time of the follow-up pouchoscopy.

**Table 3.** Comparison of clinical characteristics at the time of the index pouchoscopy in the cuffitis and/or pouchitis group vs no pouchitis or cuffitis group at the second pouchoscopy

Variable	All	Pouchitis and/or cuffitis	No pouchitis or cuffitis	*P*-value
	(*n *=* *76)	(*n *=* *35)	(*n *=* *41)	
Demographic				
Age, years				0.23
18–50	40 (52.6)	22 (62.9)	18 (43.9)	
51–70	32 (42.1)	12 (34.3)	20 (48.8)	
>70	4 (5.3)	1 (2.9)	3 (7.3)	
Male	40 (52.6)	20 (57.1)	20 (48.8)	0.47
Smoking status^a^				0.02
Non-smoker	62 (82.7)	24 (68.6)	38 (95.0)	
Former smoker	12 (16.0)	10 (28.6)	2 (5.0)	
Current smoker	1 (1.3)	1 (2.9)	0 (0.0)	
Body mass index, kg/m^2^				0.32
<18.5	7 (9.2)	5 (14.3)	2 (4.9)	
18.5–24.9	46 (60.5)	18 (51.4)	28 (68.3)	
25.0–29.9	20 (26.3)	11 (31.4)	9 (22.0)	
≥30	3 (3.9)	1 (2.9)	2 (4.9)	
Family history of IBD or colon cancer^b^	25 (34.2)	12 (35.3)	13 (33.3)	0.89
IBD information				
IBD subtype				0.96
Crohn's disease	10 (13.2)	5 (14.3)	5 (12.2)	
Ulcerative colitis	57 (75.0)	26 (74.3)	31 (75.6)	
Indeterminate colitis	9 (11.8)	4 (11.4)	5 (12.2)	
Time since IBD diagnosis^c^, years				0.41
0–10	10 (19.2)	4 (16.7)	6 (21.4)	
10–20	15 (28.8)	10 (41.7)	5 (17.9)	
20–30	14 (26.9)	6 (25.0)	8 (28.6)	
>30	13 (25.0)	4 (16.7)	9 (32.1)	
Surgical information				
J pouch^d^	71 (95.9)	33 (97.1)	38 (95.0)	0.90
Time since surgery^d^, years				0.45
0–10	25 (33.8)	12 (36.4)	13 (31.7)	
10–20	20 (27.0)	10 (30.3)	10 (24.4)	
20–30	18 (24.3)	6 (18.2)	12 (29.3)	
>30	11 (14.9)	5 (15.2)	6 (14.6)	
Surgical stage^e^				0.13
Stage 1	6 (17.6)	1 (7.7)	5 (23.8)	
Stage 2	12 (35.3)	3 (23.1)	9 (42.9)	
Stage 3	16 (47.1)	9 (69.2)	7 (33.3)	
Medically refractory disease	70 (92.1)	34 (97.1)	36 (87.8)	0.13
Medical history				
Coronary artery disease	1 (1.3)	0 (0.0)	1 (2.4)	0.35
Coronary artery bypass graft	0 (0.0)	0 (0.0)	0 (0.0)	ns
Chronic obstructive pulmonary disease	3 (3.9)	1 (2.9)	2 (4.9)	0.65
Liver cirrhosis	1 (1.3)	0 (0.0)	1 (2.4)	0.35
History of deep vein thrombosis	5 (6.6)	2 (5.7)	3 (7.3)	0.96
Chronic kidney disease/end-stage renal disease	1 (1.3)	0 (0.0)	1 (2.4)	0.35
Current medication use				
Antibiotic	19 (25.0)	7 (20.0)	12 (29.3)	0.84
Topical or oral mesalamine	14 (18.4)	6 (17.1)	8 (19.5)	0.79
Topical or oral steroids	9 (11.8)	4 (11.4)	5 (12.2)	0.92
6MP/AZA/MTX	1 (1.3)	1 (2.9)	0 (0.0)	0.46
Anti-TNFs	9 (11.8)	4 (11.4)	5 (12.2)	0.92
Vedolizumab	9 (11.8)	5 (14.3)	4 (9.8)	0.54
Ustekinumab	9 (11.8)	4 (11.4)	5 (12.2)	0.92
Other disease-modifying agents	1 (1.3)	0 (0.0)	1 (2.4)	1.00

IBD = inflammatory bowel disease; 6MP = 6-mercaptopurine; AZA = azathioprine; MTX = methotrexate; Anti-TNFs = antitumor necrosis factor; ns = not significant given small sample size.

a *n *=* *75.

b *n *=* *73.

c *n *=* *52.

d *n *=* *74.

e *n *=* *34.

### Logistic regression

Univariable and multivariable logistic regression for the primary outcome of cuffitis and/or pouchitis detected no significant associations (Table 4).

**Table 4.** Multivariable logistic regression model for the presence of cuffitis and/or pouchitis at follow-up.

Variable	Univariable		Multivariable	
	OR (95% CI)	*P*-value	OR (95% CI)	*P*-value
Mucosectomy	1.40 (0.54–3.6)	0.49	1.37 (0.48–3.9)	0.56
Smoking	0.99 (0.97–1.0)	0.71	0.99 (0.97–1.0)	0.69
Male	1.40 (0.56–3.5)	0.47	1.38 (0.54–3.5)	0.51
Use of antibiotic	1.0 (0.99–1.0)	0.92	1.0 (1.0–1.0)	0.88
Time since surgery	1.0 (0.68–1.5)	0.07	0.98 (0.64–1.5)	0.91

OR = odds ratio; CI = confidence interval.

## Discussion

This retrospective cohort study found that mucosectomy (compared to non-mucosectomy) was not associated with decreased risk for cuffitis and/or pouchitis among patients with IBD. Indeed, mucosectomy had a non-significant trend towards increased risk for cuffitis and/or pouchitis and was associated with increased likelihood of stricture formation compared with non-mucosectomy. Other outcomes related to the pouch (e.g. prolapse or the need for endoscopic pouch therapy) were similar between the mucosectomy and non-mucosectomy groups. In conjunction with other studies, this supports the use of a stapled anastomosis (non-mucosectomy) technique.

This study adds to the growing body of literature assessing the impact of mucosectomy on several clinical and functional outcomes. With regard to oncologic outcomes, a 2010 retrospective study of 3,203 patients at Cleveland Clinic concluded that mucosectomy did not protect against pouch neoplasia [11]. A retrospective study of 81 patients affirmed that, even in patients with UC with coexisting dysplasia or cancer, stapled anastomosis had a similar risk of dysplasia compared to hand-sewn anastomosis [9], although a meta-analysis suggested that there still might be a role for mucosectomy among patients with cancer or dysplasia [12].

With regard to structural and functional outcomes, a 2003 study found that post-operative complications were more common in mucosectomy patients compared with non-mucosectomy patients [13]. A 1994 retrospective cohort study of 88 patients determined that there was no difference in functional outcomes (including urgency, number of bowel movements, and use of antidiarrheals) and increased incidence of leakage and pouchitis in the mucosectomy group [14]. Another study identified mucosectomy as an independent risk factor for pouch failure [12]. Two meta-analyses have also concluded that mucosectomy does not confer benefit and that it might be associated with harm [4, 10].

While functional outcomes and dysplasia risks associated with mucosectomy have been explored in the above studies, there is less available literature on the association between the anastomosis technique and the incidence of pouchitis and cuffitis. A 2013 historical cohort study notes that a stapled J pouch without mucosectomy may increase the risk for cuffitis [2]. As previously noted, a meta-analysis speculated that mucosectomy would reduce the occurrence of cuffitis, but noted the difficulty in determining this given the common co-occurrence of this condition with pouchitis [10]. This study builds on prior findings by using a rigorous study design in which patients were required to have a normal index pouchoscopy in order to create an “even playing field” between patients who had undergone mucosectomy and those who had undergone non-mucosectomy at the start of the study; this important feature of the study design has been lacking from some prior studies that were either cross-sectional or did not fully account for baseline differences between groups.

Interestingly, smoking was the only factor associated with the presence of cuffitis and/or pouchitis at follow-up pouchoscopy. The literature remains conflicted on the effect of smoking on complications in patients with IPAA. One study suggests that smoking may be protective against pouchitis while simultaneously increasing the risk of Crohn’s disease of the pouch [15]. While our study did not support this conclusion, our sample size is smaller than that of the aforementioned paper and this was not the focus of our investigation. Another notable finding from our study was a statistically significant association between stricture and mucosectomy. This phenomenon has been previously documented, with one study noting a stricture incidence of 24% in patients with UC with hand-sewn anastomosis [16]. An additional study noted a significantly higher incidence of strictures in hand-sewn anastomosis compared with stapled anastomosis [17]. This complication often requires endoscopic therapy [18]. However, the literature remains conflicted on the strength of this association and does not provide a clear pathogenesis for this occurrence. Our study also found an association between time since surgery, as well as disease duration, and mucosectomy, possibly reflective of the previously prevalent sentiment that mucosectomy was protective against neoplasia, which has been refuted in more recent literature as described above.

Our study does have several limitations. It was conducted at a single, highly specialized referral center with a relatively small sample size, as evidenced by the unusually high incidence of pouch prolapse in our cohort. This may impact the generalizability of our findings to other centers and populations. Additionally, the retrospective nature of the study may introduce biases, including missing data. The primary outcome was based on endoscopic findings rather than a more patient-centered outcome based on symptoms. Additional important limitations include lack of histology in the majority of patients, a compressed follow-up time period, and possible selection bias because patients who were treated empirically for pouchitis were not included in the study. Further, the original operative reports were not always available for review; rather, the presence or absence of mucosectomy was based on clinical documentation and on endoscopic review by one of the authors (B.S.). Given this, the variability of endoscopy quality should also be noted as a limitation. An additional limitation is that the indication for the index pouchoscopy was unknown, although it was assumed that most of these normal pouchoscopies were being done for routine surveillance for dysplasia. The study also has some strengths. Namely, we retrospectively followed patients for ≤1 year and used a time-to-event analysis, as opposed to many prior studies on this topic. Further, we examined multiple outcomes related to inflammation, giving a more comprehensive picture of the inflammatory effects after IPAA. We also assessed these outcomes using a validated scale, providing a more standardized measure of inflammatory outcomes.

## Conclusions

Mucosectomy (hand-sewn anastomosis) was not found to be associated with reduced risk of cuff or pouch inflammation compared to non-mucosectomy (stapled anastomosis). Patients with hand-sewn anastomoses were more likely to experience strictures than those with stapled non-mucosectomy anastomoses. These results, in conjunction with prior research, may support the selection of stapled anastomosis over hand-sewn anastomosis with mucosectomy as the preferred surgical method for patients with IBD undergoing colectomy with IPAA.

## Supplementary Data

Supplementary data is available at *Gastroenterology Report* online.

## Authors’ Contributions

A.H., B.S., and D.F. made substantial contributions to the conception and design of the study; acquisition, analysis, and interpretation of data; and drafting of the article, as well as critical revision of important intellectual content. All authors have read and approved the final version of the manuscript. B.S. and D.F. contributed equally and are designated co-corresponding authors.

## Supplementary Material

goad078_Supplementary_Data
