# Supplementary material for: Association between mucosectomy and endoscopic outcomes in patients with ileal pouch–anal anastomosis
Source: Gastroenterol Rep (Oxf). 2024 Jul 4;12:goad078. doi: 10.1093/gastro/goad078 (PMC11222711; doi:10.1093/gastro/goad078)
Supplement: goad078_Supplementary_Data [file goad078_supplementary_data.zip › Supplementary data.docx]

**Supplementary data**

**Supplementary Table 1.** Relationship between mucosectomy and cuffitis and/or pouchitis, stratified by exposure to antibiotics.

| Item | Antibiotics (*n* = 19) | | |  | No antibiotics (*n* = 57) | | |
| --- | --- | --- | --- | --- | --- | --- | --- |
|  | Presence of cuffitis and/or pouchitis | Absence of cuffitis or pouchitis | *P*-value |  | Presence of cuffitis and/or pouchitis | Absence of cuffitis or pouchitis | *P*-value |
| Mucosectomy |  |  | 0.31 |  |  |  | 0.93 |
| Yes | 4 (57.1) | 4 (33.3) |  |  | 20 (71.4) | 21 (72.4) |  |
| No | 3 (42.9) | 8 (66.7) |  |  | 8 (28.6) | 8 (27.6) |  |

**Supplementary Figure 1.** Study timeline schematic. Patients were eligible for the study if they had an index pouchoscopy that was normal (i.e., no cuffitis or pouchitis) between 2020 and 2022 and then a second follow-up pouchoscopy within the same time period. They were retrospectively followed up from this first index pouchoscopy to the second pouchoscopy for the primary outcome (present of cuffitis and/or pouchitis). No specific interval was required between proctocolectomy and the index pouchoscopy.
