# Supplementary figures and images for: Association between mucosectomy and endoscopic outcomes in patients with ileal pouch–anal anastomosis
Source: Gastroenterol Rep (Oxf). 2024 Jul 4;12:goad078. doi: 10.1093/gastro/goad078 (PMC11222711; doi:10.1093/gastro/goad078)

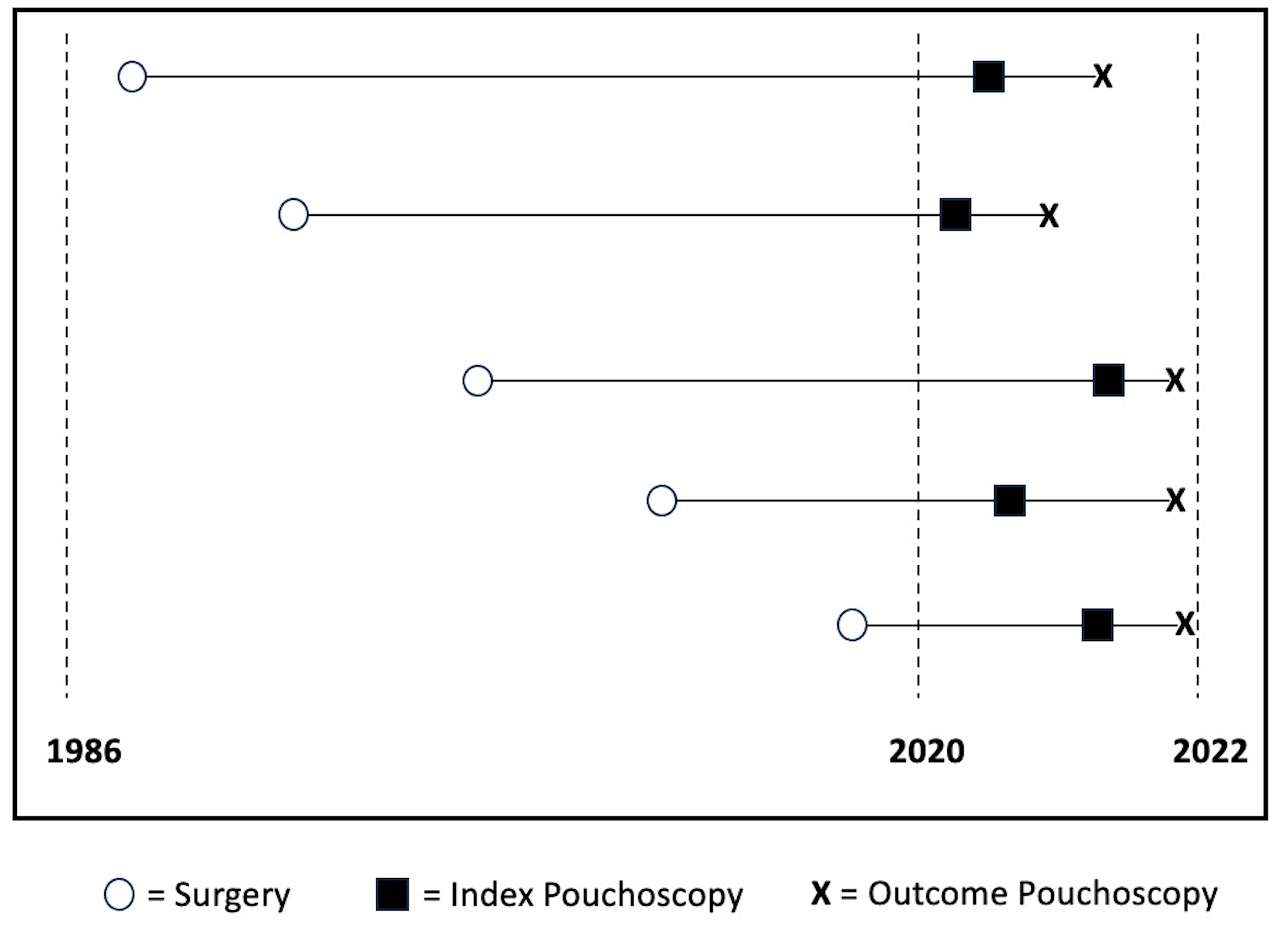

Supplement: goad078_Supplementary_Data [file goad078_supplementary_data.zip › Supplementary Fig 1 final version.jpeg]
